# RING-finger type E3 ubiquitin ligase inhibitors as novel candidates for the treatment of rheumatoid arthritis

**DOI:** 10.3892/ijmm.2012.1129

**Published:** 2012-09-18

**Authors:** NAOKO YAGISHITA, SATOKO ARATANI, CRAIG LEACH, TETSUYA AMANO, YOSHIHISA YAMANO, KO NAKATANI, KUSUKI NISHIOKA, TOSHIHIRO NAKAJIMA

**Affiliations:** 1 Institute of Medical Science, St. Marianna University School of Medicine, Kawasaki, Kanagawa;; 2 Institute of Medical Science, Tokyo Medical University, Shinjuku, Tokyo, Japan;; 3 Progenra, Inc., Malvern, PA, USA;; 4 Bayside Misato Medical Center, Kochi;; 5 Choju Medical Institute Fukushimura Hospital, Toyohasi, Japan

**Keywords:** rheumatoid arthritis, synoviolin, E3 ubiquitin ligase, endoplasmic reticulum associated degradation, inhibitor

## Abstract

Rheumatoid arthritis (RA) significantly affects quality of life. We recently cloned synoviolin, a RING-type E3 ubiquitin ligase implicated in the endoplasmic reticulum-associated degradation (ERAD) pathway. Synoviolin is highly expressed in rheumatoid synovial cells and may be involved in the pathogenesis of RA. Inhibition of synoviolin activity is a potentially useful therapeutic approach for the treatment of RA. We conducted a high-throughput screen of small molecules to find inhibitors of synoviolin autoubiquitination activity. We identified two classes of small molecules, named LS-101 and LS-102, which inhibited synoviolin activity. LS-102 selectively inhibited synoviolin enzymatic activity, while LS-101 inhibited a broad array of RING-type E3 ligases. Moreover, these inhibitors suppressed the proliferation of rheumatoid synovial cells, and significantly reduced the severity of disease in a mouse model of RA. Our results suggest that inhibition of synoviolin is a potentially useful approach in the treatment of RA.

## Introduction

Rheumatoid arthritis (RA) is the most common chronic inflammatory joint disease, affecting ∼0.5–1% of people in the industrialized world (1). Clinically, the disorder is characterized by joint pain, stiffness, and swelling due to synovial inflammation and effusion. The clinical features of RA are based on several pathological processes including chronic inflammation, overgrowth of synovial cells, bone and joint destruction, and fibrosis. Currently, the goal of RA treatment is the control of underlying inflammatory process to prevent joint damage using non-steroidal anti-inflammatory drugs, glucocorticoids, and disease-modifying anti-rheumatic drugs (DMARD). The most widely used small molecule DMARD is methotrexate, which shows the highest retention rate compared with other agents (2). In recent years, biological agents such as inhibitors of tumor necrosis factor (TNF) signaling have become available for clinical use; however, this therapy is prohibitively expensive, and although TNF inhibitors are clinically as effective as methotrexate, the frequency and extent of response are more restricted. In fact, many patients can lose the clinical response to TNF inhibition, highlighting the need for other treatment modalities to further improve the outcome of RA (3,4).

To address this need, we have been investigating the mechanism of outgrowth in rheumatoid synovial cells (RSCs). First, we demonstrated the crucial role of Fas antigen-induced apoptosis in synovial cell hyperplasia (5). Then, while studying cellular functions of RSCs, we cloned synoviolin from these cells (6). Synoviolin, a mammalian homolog of Hrd1p/Der3p (7–9), is an endoplasmic reticulum (ER)-resident E3 ubiquitin ligase with a RING motif that is involved in ER-associated degradation (ERAD) pathway. Synoviolin is also highly expressed in synoviocytes of patients with RA (6,10–12). Overexpression of synoviolin in transgenic mice leads to advanced arthropathy caused by reduced apoptosis of synoviocytes (6). We postulated that hyperactivation of the ERAD pathway by overexpression of synoviolin prevents ER-stress-induced apoptosis, leading to synovial hyperplasia (13). Synoviolin^+/−^ knockout mice showed resistance to the development of collagen-induced arthritis (CIA) due to enhanced apoptosis of synovial cells (6). Consistent with our hypothesis, cells from these mice show impaired ERAD due to the lack of synoviolin. In addition, synoviolin ubiquitinates and sequesters the tumor suppressor p53 in the cytoplasm, thereby negatively regulating its biological functions in transcription, cell cycle regulation, and apoptosis by targeting it instead for proteasomal degradation (14). Therefore, synoviolin regulates apoptosis in response to ER stress (through ERAD) as well as p53-dependent apoptosis.

Together, these studies implicated synoviolin as a candidate pathogenic factor in arthropathy, and suggested that the gene dosage of this protein correlates with the onset of arthropathy. Furthermore, elevated synoviolin levels were identified in circulating monocytes in association with resistance to treatment with infliximab (a monoclonal antibody against TNF) (10). Therefore, blocking the function of synoviolin could be clinically beneficial in RA patients. This study attempted to identify an inhibitor of synoviolin that acts by blocking its enzymatic activity.

## Materials and methods

### Screening of synoviolin inhibitor

Purified glutathione S-transferase (GST)-synoviolin Δ transmembrane domain (TM) was mixed with glutathione-SPA beads (Amersham Pharmacia Biotech) in buffer (50 mM Tris-HCl, pH 7.4, Protease inhibitor cocktail, 14 mM β-mercaptoethanol, 0.5 μl cell lysate/well, 0.2 mg SPA bead/well) and incubated for 30 min at room temperature. Glutathione-SPA beads were washed twice, and then mixed with the candidate synoviolin inhibitor compounds in buffer (50 mM Tris-HCl, pH 7.4, 5 mM MgCl_2_, 2 mM NaF, and 10 nM okadaic acid) in the presence of ATP (2 mM), ^33^P-labeled ubiquitin (0.38 μg/well), E1 (25 ng/well) (Affiniti Research), and E2 (0.3 μg/well) (UbcH5c). After incubation for 90 min at room temperature, buffer comprising 0.2 M boric acid, pH 8.5, 2 mM ethylenediaminetetraacetic acid (EDTA), and 2% Triton-X100 was added to stop the reaction. The beads were allowed to settle and the amount of ^33^P-ubiquitin incorporated into the GST-synoviolin beads was determined using a Microbeta Scintillation counter.

The primary screen was conducted with multiple compounds per well (10–20 compounds per well) at an estimated screening concentration of 2–10 μM. Compound mixtures showing potential activity in the primary screen were then rescreened at one compound per well to determine the active compound within the mixture. Three equivalents of a single compound per well follow-up screening were evaluated. Reconfirmed active compounds were resynthesized and tested in a dose-response experiment to determine potency.

### In vitro ubiquitination assay

The *in vitro* ubiquitination assay used in this study was described previously (15). Briefly, 40 ng of E1 (Affiniti Research), 0.3 μg of E2 (UbcH5c), 0.75 μg of ^32^P-labeled ubiquitin (a gift from T. Ohta), and 1 μg of recombinant E3 ubiquitin ligases were incubated for 30 min at 37°C. Samples were analyzed as described above.

### Cells

HeLa cells were obtained from ATCC. Synovial cells were isolated from synovial tissue obtained patients with rheumatoid arthritis (RA) who met the American College of Rheumatology criteria for RA at the time of orthopedic surgery. These cells were cultured in Dulbecco’s modified Eagle’s medium (Sigma).

### Proliferation assay

The proliferation of rheumatoid synovial cells (RSCs) was evaluated using Alamar blue (BioSource International) according to the manufacturer’s instructions.

### Induction of CIA

CIA was induced as described previously (6). Briefly, bovine type II collagen (Collagen Research Center) was dissolved overnight in 0.05 M acetic acid at 4°C, and then emulsified in complete Freund’s adjuvant (Difco) to a final concentration 1 mg/ml. DBA/1 male mice (7-week-old) were immunized by subcutaneous injections containing 100 μg of collagen emulsion. After 3 weeks, mice were boosted with 200 μg collagen emulsion in Freund’s complete adjuvant. Then, the mice were treated daily for 4 weeks with the inhibitor compounds at 1.3, 4.0, and 12.0 mg/kg/day in olive oil, vehicle control intraperitoneally, or oral administration of 0.25 mg/kg/day dexamethasone in methylcellulose as a positive control.

The mice were monitored daily for signs of arthritis using an established scoring system (16): 0, no swelling or redness; 1, swelling, redness of paw or 1 joint; 2, two joints involved; 3, more than two joints involved; 4, severe arthritis of entire paws and joints. All paws were evaluated in each animal and the maximum score per animal was 16.

### Histological studies

The knee and elbow joints were fixed in 4% paraformaldehyde. After decalcification with EDTA, the joints were embedded in paraffin, and 4-μm sections were prepared for staining with hematoxylin and eosin. The extent of arthritis in the joints was assessed according to the method reported by Tomita *et al*(17): 0, normal synovium; 1, synovial membrane hypertrophy and cell infiltration; 2, pannus and cartilage erosion; 3, major erosion of cartilage and subchondral bone; 4, loss of joint integrity and ankylosis.

### Statistical analysis

All data are expressed as mean ± SEM. Differences between groups were examined for statistical significance using Student’s t-test. A P-value <0.05 denoted the presence of a statistically significant difference.

### Ethical considerations

The ethics committee for Animal Experiments of St. Marianna University School of Medicine approved the mice experiments described in this study. Furthermore, all the experimental protocols described in this study were approved by the Ethics Review Committee of St. Marianna University School of Medicine (Approval number 01008), and the written informed consent was obtained from all patients.

## Results

### High-throughput compound screening for inhibitors of synoviolin

**Figure 1:**
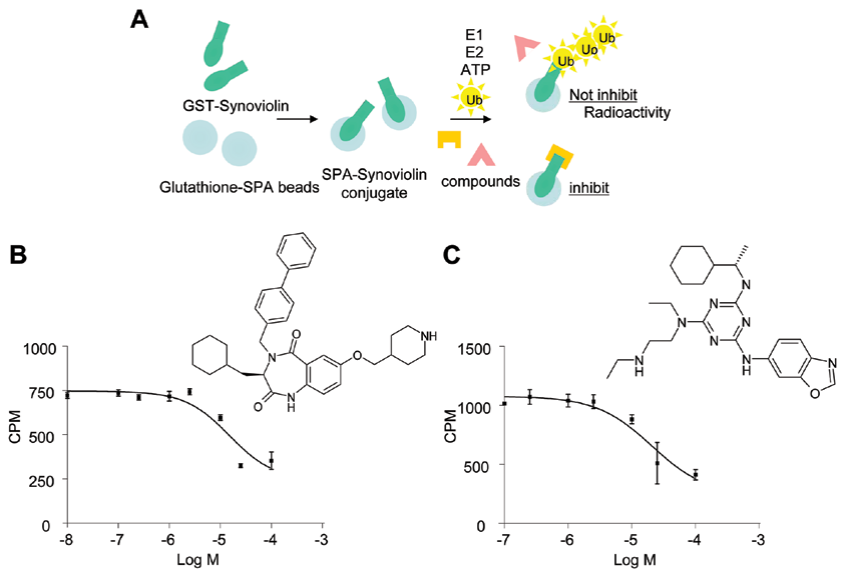
Screening for synoviolin inhibitors. (A) Scheme of high-throughput screening of synoviolin-induced ubiquitination assay. (B) Inhibition of synoviolin ^33^P-polyubiquitination by LS-101 and chemical structure of LS-101. (C) Inhibition of synoviolin ^33^P-polyubiquitination by LS-102 and chemical structure of LS-102.

To identify small molecule inhibitors of synoviolin autoubiquitination, we screened the Lead Discovery Service program of Pharmacopeia, which includes more than four million compounds from Pharmacopeia’s Compound Collection (18). Herein we monitored ^33^P-autoubiquitinated synoviolin in cell lysates containing GST-synoviolinΔTM in the presence of ATP, E1, E2, and ^33^P-labeled ubiquitin (Fig. 1A). The primary screen was conducted with multiple compounds per well (10–20 compounds per well) at an estimated screening concentration of 2–10 μM. Mixtures of compounds showing potential activity in the primary screen were then rescreened individually. Compounds demonstrating activity in this reconfirmation assay were resynthesized and retested. Two unique compounds, termed LS-101 and LS-102, inhibited the autoubiquitination of synoviolin with a 50% inhibitory concentration value (IC_50_) of ∼15 μM (Fig. 1B) and 20 μM (Fig. 1C), respectively.

### LS-101 and LS-102 inhibit the autoubiquitination of synoviolin

**Figure 2:**
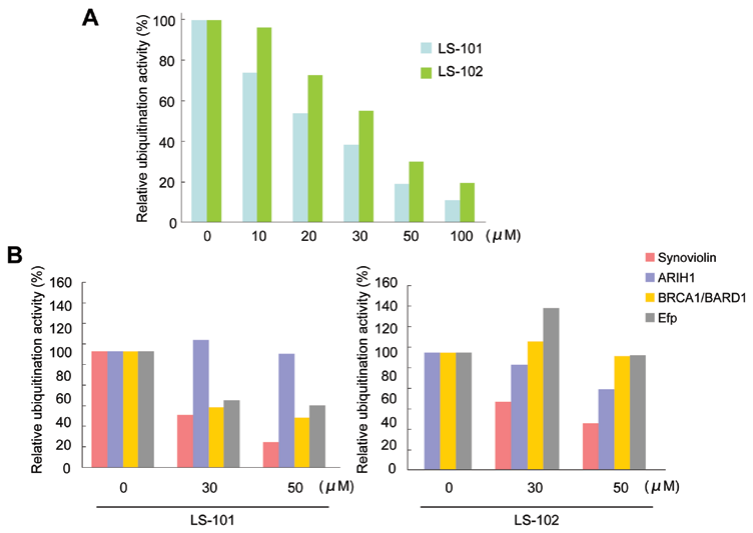
Effects of LS-101 and LS-102 on *in vitro* ubiquitination. (A) Both LS-101 and LS-102 inhibited the autoubiquitination of synoviolin in a dose-dependent manner. The IC_50_ of LS-101 was 20 μM and that of LS-102 was 35 μM. (B) Selectivity of LS-101 (left) and LS-102 (right) against other E3 ubiquitin ligases. LS-102 inhibited synoviolin selectively compared with LS-101. Data are mean ± SEM of 3 experiments.

Further evaluation of LS-101 and LS-102 in an *in vitro* ubiquitination assay showed that the inhibition of synoviolin activity by both LS-101 and LS-102 was dose-dependent (LS-101; IC_50_=20 μM, LS-102; IC_50_=35 μM) (Fig. 2A). To assess the selectivity of the compounds for other E3 ubiquitin ligases, we determined the effects of LS-101 and LS-102 on the enzymatic activity of the following RING-finger type E3 ubiquitin ligases: ariadne, *Drosophila*, homolog of, 1 (ARIH1) (19), breast cancer 1 gene (BRCA1)/BRCA1-associated RING domain 1 (BARD1) (20), and estrogen-responsive RING-finger protein (Efp) (21). LS-101 inhibited the activity of BRCA1/BARD1 and Efp (Fig. 2B), although this effect was weaker than that observed with synoviolin (Fig. 2B). Moreover, LS-101 had no effect against the enzymatic activity of ARIH1 (Fig. 2B). On the other hand, LS-102 did not inhibit the activity of other E3 ubiquitin ligases, only affecting synoviolin (Fig. 2B). These results suggested that LS-102 is a more selective synoviolin inhibitor than LS-101.

### LS-101 and LS-102 inhibit proliferation of RSCs

**Figure 3:**
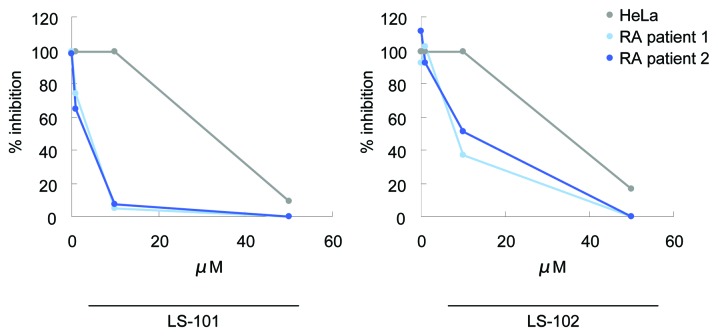
Effects of LS-101 and LS-102 on cell growth of RSCs. HeLa cells and RSCs derived from two RA patients were treated with synoviolin inhibitors for 12 h at the indicated concentrations. LS-101 and LS-102 repressed the proliferation of each RSC population tested. Data are expressed as the mean percentage of inhibition of the vehicle-treated control group ± SEM; (n=3).

We next tested LS-101 and LS-102 for their effects on the proliferation of RSCs, using HeLa cells as a control. LS-101 and LS-102 inhibited HeLa cell growth only at very high concentrations (LS-101; IC_50_=31.3 μM, LS-102; IC_50_=32.7 μM). However, treatment of RSCs with these compounds suppressed synovial cell growth dose-dependently and with much greater potency than that observed in HeLa cells (Fig. 3). A similar effect was also observed in another line of RSCs (Fig. 3). In addition, LS-101 inhibited synovial cell proliferation more potently than LS-102 (LS-101; IC_50_=4.2 μM, LS-102; IC_50_=5.4 μM). These results demonstrated that blockade of synoviolin function reduced the proliferation of RSCs, and that RSCs are more susceptible to this effect than HeLa cells. Consistent with these findings, higher expression levels of synoviolin were observed in RSCs than in HeLa cells (6).

### LS-101 and LS-102 reduce clinical severity scores in a CIA model

**Figure 4:**
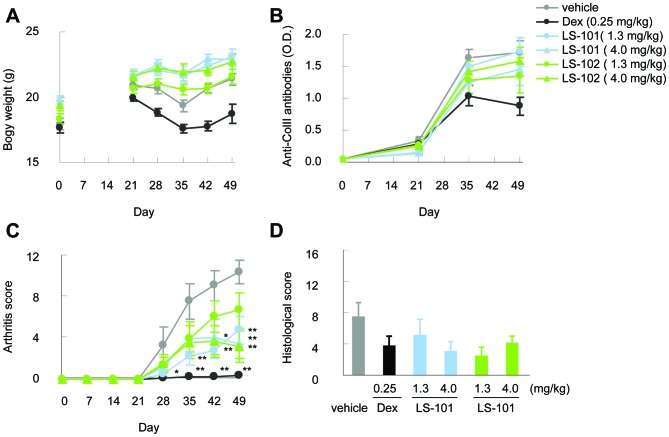
Effects of LS-101 and LS-102 in mouse CIA. DBA/1 mice immunized on day 0 and boosted on day 21 with type II collagen were treated with the vehicle alone, 0.25 mg/kg dexamethasone (Dex), or with 1.3, 4.0 mg/kg LS-101 or LS-102 from day 21 to 49. (A) Change in body weight. (B) The level of anti-type II collagen antibodies. (C) Total arthritis score. (D) Histological arthritis score. Data are mean ± SEM (initial n=12; final n=7). ^*^P<0.05, ^**^P<0.01.

To evaluate the *in vivo* efficacy of synoviolin inhibitors, we tested LS-101 and LS-102 in a mouse model of arthritis over a period of 28 days. No reduction of body weight was observed during the administration of these compounds (Fig. 4A). Moreover, the production of anti-type II collagen antibodies resulting from type II collagen immunization in both the LS-101 and LS-102 group was comparable to that observed in the vehicle control group (Fig. 4B). Intraperitoneal treatment with LS-101 or LS-102 starting on day 21 reduced the clinical severity scores compared to vehicle controls (Fig. 4C). The efficacy was observed at both 1.3 mg/kg and 4.0 mg/kg doses in this experiment, although the protective effect of LS-101 at 1.3 mg/kg against CIA was stronger than the same dose of LS-102. At 4.0 mg/kg, there was no difference in the effects between LS-101 and LS-102. Finally, histological analysis showed lower histological arthritis scores in mice treated with the synoviolin inhibitors compared with wild-type mice (Fig. 4D).

## Discussion

The selective degradation of proteins in eukaryotic cells is carried out by the ubiquitin proteasome system (UPS), whereby proteins are targeted for degradation by covalent ligation to small polypeptide ubiquitin (22,23). This reaction requires the sequential actions of three enzymes: E1, E2, and E3 ligases (22,23). E3 ligases are responsible for conferring selectivity to ubiquitination by recognizing specific substrates. Bioinformatic analysis has identified over 600 E3 ligases, with RING-type E3 ligases constituting the largest subfamily within this group (24). Accordingly, RING E3 ligases have been linked to the control of multiple cellular processes and to many human diseases such as diabetes mellitus, polyglutamine disease, and Parkinson’s diseases (24–26). In the UPS, the proteasome inhibitory agent bortezomib (Velcade) was recently approved for the treatment of multiple myeloma and mantle cell lymphoma (27). Bortezomib induces apoptosis of a wide variety of cancer cells, and is the first proteasome inhibitor to gain FDA approval (28–30). However, widespread clinical use of bortezomib continues to be hampered by the appearance of dose-limiting toxicities, drug-resistance, and interference by some natural compounds (31). Thus, despite the efficacy of bortezomib for treating lethal diseases such as cancer, the associated toxicities prevent its use for the treatment of chronic diseases such as RA. Thus, it is important to develop inhibitors of the ubiquitin-proteasome enzymatic cascade upstream from the proteasome to impact fewer cell processes and reduce toxicity. E3 ligases are attractive such targets given their large number and substrate specificity. We recently cloned the E3 ubiquitin ligase synoviolin, which localizes to the ER lumen and has enzymatic activity. We have also demonstrated that this protein plays crucial roles in the pathological processes of RA (6), and could therefore be a candidate novel therapeutic target of RA (32).

In this study, we identified two potent small compounds as inhibitors of synoviolin enzymatic activity using high-throughput screening (Fig. 1). Moreover, *in vivo* studies showed no serious toxicity associated with these compounds in terms of survival and weight loss during treatment (Fig. 4A). Biochemical characterization of the two compounds, LS-101 and LS-102, demonstrated that they both inhibit the autoubiquitination activity of synoviolin *in vitro* (Fig. 2), with LS-101 showing stronger efficacy (IC_50_=20 μM) than LS-102 (IC_50_=35 μM), but less selectivity (Fig. 2). It was unclear from this study why LS-101 showed a weak inhibitory effect on BRCA1/BARD1 and Efp activity, and further study is needed to understand the molecular basis for this observation. LS-101 and LS102 inhibited the proliferation of RSCs and to a much lesser extent, HeLa cells (Fig. 3). The difference in cell sensitivities to these compounds could be, at least in part, due to the expression level of synoviolin, namely, high levels of synoviolin in RSCs would contribute to the cell overgrowth and therefore, inhibition of synoviolin in these cells would in turn suppress proliferation. These cells may also have different requirements for synoviolin, such that repressing synoviolin activity in RSCs would lead to growth suppression. Prophylactic administration of either LS-101 or LS-102 also significantly reduced the severity of murine CIA (Fig. 4C). Since LS-101, a nonselective inhibitor, reduced clinical severity scores in CIA similarly to LS-102, blocking synoviolin enzymatic activity seems crucial in the pathological process of CIA. These findings suggest that the suppression level of synovial cell growth and incidence of arthritis reflect the efficacy of these compounds rather than their selectivity, and that in RA, synoviolin might have an indispensable role among E3 ligases.

RA comprises multiple processes such as chronic inflammation, overgrowth of synovial cells, joint destruction, and fibrosis. During the course of inflammation, synovial cells, macrophages, T cells, and B cells all contribute to the production of cytokines such as interleukin (IL)-1, IL-6, IL-10, TNF, and transforming growth factor β (TGF-β) (33,34). These cytokines, in turn, stimulate the overgrowth of synovial cells to form a mass of synovial tissue, called pannus, which invades and destroys the bone and cartilage through osteoclast activation and protease production (33–37). This chronic inflammation state ultimately leads to fibrosis. Our study proved that synoviolin is, at least in part, involved in the overgrowth of synovial cells (6) and fibrosis (38) among these processes. The IL-17 induction of synoviolin may also contribute to RA chronicity (39), and synoviolin has been shown to target misfolded MHC class I heavy chains (40). In this study, antibody titers were elevated in synoviolin inhibitor-treated mice to levels comparable to those in vehicle controls (Fig. 4B). Thus, as with the study of synoviolin^+/−^ knockout mice in CIA, it is difficult to clarify the function of synoviolin with respect to the chronicity of inflammation, because suppressing synoviolin blocks synovial cell outgrowth directly due to sequential events following immunization of type II collagen (6). Our results confirm that further studies of the association between chronic inflammation and synoviolin are clearly warranted.

Eight biological agents are currently approved for clinical use in treatment of RA, and these drugs have dramatically changed the outcome of RA during the past decade (3,4). However, some patients still fail to respond to the biological treatment or develop adverse effects such as an increased risk of infection. Moreover, these agents are associated with high costs and discomfort arising from the subcutaneous or intravenous administration. Thus, there is a clear need for the development of cheaper, orally administered therapies with fewer side effects. In this regard, spleen tyrosine kinase (Syk) inhibitor, an orally administered drug, has been developed for the treatment of RA (41,42). Dual blockade of TNF and IL-17 was also reported recently as a strategy for halting RA disease from progression to the extent seen when only one cytokine is blocked (43). The involvement of synoviolin in both the TNF and IL-17 pathways further implicates inhibitors of this enzyme as potential candidate drugs for treatment of RA.

In conclusion, we identified two strong synoviolin inhibitors, and confirmed that synoviolin is an ideal molecular target for RA for disease modification and treatment. We are now proceeding with the optimization of LS-101 and LS-102, and hope our research will lead to the development of a new therapy for RA.
